# KLF7-regulated ITGA2 as a therapeutic target for inhibiting oral cancer stem cells

**DOI:** 10.1038/s41419-025-07689-8

**Published:** 2025-05-02

**Authors:** Xin Qi, Jiang Zhou, Pan Wang, Yunyan Li, Haoran Li, Yuwen Miao, XiaoQing Ma, Xiayan Luo, Zhiling Zhang, Yanling He, Wenyi Shen, Wenquan Zhao, Rutao Cui, Cang Li, Huiyong Zhu, Jiong Lyu

**Affiliations:** 1 Zhejiang University, School of Medicine, First Affiliated Hospital, Hangzhou, Zhejiang P. R. China; 2 Cancer Institute (Key Laboratory of Cancer Prevention and Intervention, China National Ministry of Education), The Second Affiliated Hospital, Zhejiang University School of Medicine, Hangzhou, Zhejiang China; 3 Zhejiang Provincial Clinical Research Center for CANCER; Cancer Center of Zhejiang University, Hangzhou, China; 4 Department of Stomatology, Children’s Hospital, Zhejiang University School of Medicine, National Clinical Research Center for Child Health, Hangzhou, China; 5 Zhejiang University, School of Medicine, Affiliated Stomatology Hospital, Hangzhou, Zhejiang P. R. China

**Keywords:** Cancer stem cells, Cancer therapeutic resistance

## Abstract

Cancer stem cells (CSCs) play crucial roles in tumor metastasis, therapy resistance, and immune evasion. Identifying and understanding the factors that regulate the stemness of tumor cells presents promising opportunities for developing effective therapeutic strategies. In this study on oral squamous cell carcinoma (OSCC), we confirmed the key role of KLF7 in maintaining the stemness of OSCC. Using chromatin immunoprecipitation sequencing and dual-luciferase assays, we identified ITGA2, a membrane receptor, as a key downstream gene regulated by KLF7 in the maintenance of stemness. Tumor sphere formation assays, flow cytometry analyses, and in vivo limiting dilution tumorigenicity evaluations demonstrated that knocking down ITGA2 significantly impaired stemness. Upon binding to its extracellular matrix (ECM) ligand, type I collagen, ITGA2 activates stemness-associated signaling pathways, including PI3K-AKT, MAPK, and Hippo. TC-I 15, a small-molecule inhibitor of the ITGA2-collagen interaction, significantly sensitizes oral squamous cell carcinoma (OSCC) to cisplatin in xenograft models. In summary, we reveal that the KLF7/ITGA2 axis is a crucial modulator of stemness in OSCC. Our findings suggest that ITGA2 is a promising therapeutic target, offering a novel anti-CSC strategy.

## Introduction

Oral cancer affects the buccal mucosa, floor of the mouth, oral tongue, alveolar ridge, retromolar trigone, and hard palate. It has an incidence rate of approximately 1 in 100,000 individuals [1]. The most common pathological type is oral squamous cell carcinoma (OSCC), accounting for more than 90% of cases [2]. Early-stage OSCC (Stages I–II) is typically treated with surgery alone, whereas advanced OSCC (Stages III-IV) requires multimodal treatment approaches and has a relatively poor prognosis due to its tendency for recurrence and lymph node metastasis [3]. Due to low early diagnosis rates, most OSCC patients are already at an advanced stage at the time of diagnosis [4]. Current treatments for advanced OSCC primarily include surgery, chemotherapy, radiotherapy, or a combination of these approaches. Despite the various treatments employed over the decades, the overall survival rate for OSCC remains at 50% [5]. Common chemotherapy agents for OSCC include platinum drugs, 5-fluorouracil (5-FU), paclitaxel (PTX), and doxorubicin (Dox). However, the majority of patients develop resistance to these drugs. At present, multidrug resistance is one of the primary obstacles to successful cancer chemotherapy and leads to poor patient prognosis [6].

The concept of cancer stem cells (CSCs) has evolved over several decades [7]. They were first identified in the 1990s in hematologic malignancies and have since been isolated from various hematologic and solid tumors, including oral cancer [8–13]. Oral cancer stem cells (OCSCs) have the capacity for long-term self-renewal and can replicate the diverse cell lineages found in primary cancers [14, 15]. One characteristic of OCSCs is their propensity to develop resistance to treatments, including both conventional chemotherapy and immunotherapy. Therefore, therapies targeting OCSCs hold promise as an effective approach to overcoming resistance in OSCC. Developmental signaling pathways, including Hippo, Notch, and WNT, are frequently altered in CSCs and play key regulatory roles in supporting stem cell maintenance and survival [16]. Various CSC-targeted therapies have been developed and are currently in clinical trials, such as NEDD8-activating enzyme inhibitors targeting the Hippo pathway, γ-secretase inhibitors targeting the Notch pathway, and small-molecule antagonists of β-catenin targeting the WNT pathway [17–21]. Progress has also been made in targeting OCSCs in oral cancer. For example, Li et al. demonstrated that β-catenin silencing enhances OSCC sensitivity to cisplatin [22]. Additionally, CD133 expression is elevated in OSCC and associated with increased resistance; targeting CD133 combined with cisplatin treatment effectively inhibits OCSC-driven OSCC initiation [23].

Utilizing novel drugs to target therapy-resistant CSCs may reduce cancer recurrence rates and improve treatment efficacy. To mitigate drug resistance in OSCC, it is essential to improve knowledge of OCSCs, with a particular focus on molecular features. Recent evidence suggests that CSCs represent a plastic cellular state influenced by dynamic interactions within the CSC niche, rather than a fixed condition [24]. Differentiated cancer cells can revert to a more undifferentiated state under certain conditions or stimuli [25]. The maintenance of CSCs depends on the tumor microenvironment and niche. In this study, we employed single-cell analysis to identify a stem-like subset within OSCC, characterized by high developmental potential and activation of stemness pathways. We identified KLF7 as a potential key molecule in maintaining OCSC. Further investigation revealed that KLF7 regulates OSCC stemness through the direct transcriptional activation of ITGA2, a membrane protein crucial for mediating cell-ECM interactions. ITGA2 inhibition can suppress OSCC stemness and significantly sensitizes OSCC to cisplatin. Our findings highlight that ITGA2 is a promising therapeutic target, offering a novel anti-CSC strategy.

### Results

#### Identification of CSC and key molecules for maintaining stemness in OSCC

**Fig. 1:**
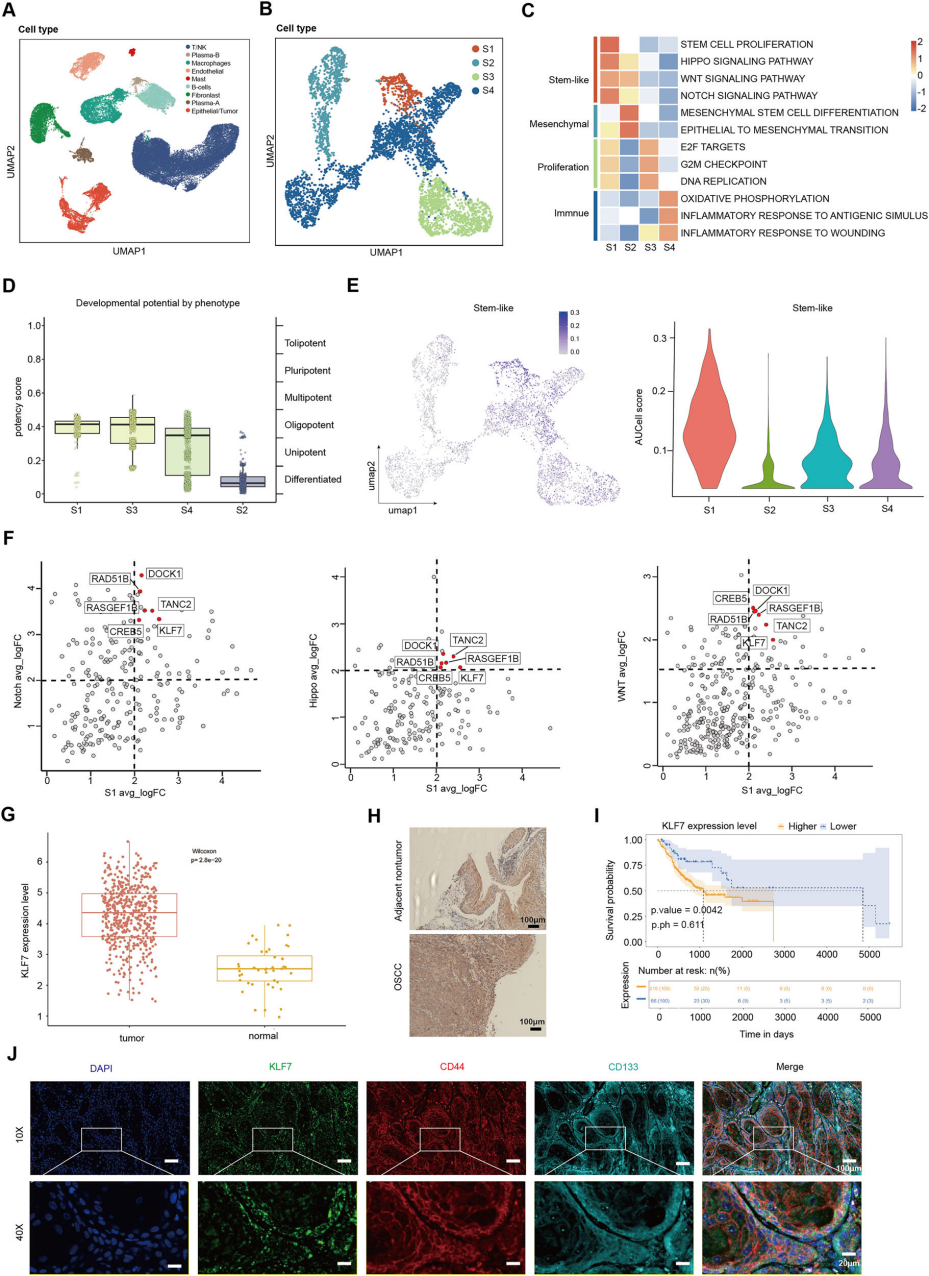
Identification of cancer stem cells and key molecules for maintaining stemness in oral cancer. **A** UMAP illustrating cell type diversity in human OSCC (55k cells). **B** UMAP plot of the subtype of malignant cells analyzed by scRNA-seq. **C** Heatmap depicting enriched pathway of the subtype of malignant cells. **D** CytoTRACE plot of the subtype of malignant cells. **E** Stem-like gene sets activity (AUCell score) in all malignant cells (umap plots, left panel) or per transcriptional state (violin plots, right panel). **F** The log2-transformed fold change (FC) in RNA levels between the stem-like cluster with the rest of the clusters and the high enrichment in the stem-related pathway. **G** KLF7 expression in OSCC and normal samples. **H** Representative immunohistochemistry image showing the expression of KLF7 in human OSCC tissue. Scale bar, 100 μm. **I** Kaplan–Meier curve of KLF7 in OSCC TCGA dataset. **J** Representative immunofluorescent image showing the expression of KLF7, CD44, CD133, and DAPI in human OSCC tissue. Scale bar, 100 μm (top); Scale bar, 20 μm (bottom).

To identify the key molecule of CSCs in OSCC, we analyzed single-cell transcriptome data from 10 OSCC samples obtained from public databases (Supplementary Fig. 1A and Supplementary Table S1). After a series of preprocessing steps, we annotated 55,000 cells using previously published marker gene sets [26–28] (Fig. 1A and Supplementary Fig. 1B). We Used HoneyBadger to exhibit high copy-number variation (CNV) (Supplementary Fig. 1C). Epithelial-derived cells with CNV > 0.1 were identified as malignant cells and further subdivided into subtypes. Unsupervised Louvain-based clustering and UMAP identified four distinct OSCC cell states, Gene Set Variation Analysis (GSVA) functionally annotated each cluster, and S1 was identified as the stem-like subtype (Fig. 1B, C and Supplementary Fig. 1D and Supplementary Table S2). S1 exhibited the highest developmental potential, positioned at the beginning of the cell trajectory, and showed the highest stemness gene expression activity [29] (Fig. 1D, E, Supplementary Fig. 1E, and Supplementary Table S3). Consistent with studies linking CSCs and tumor metastasis [30], we found that S1 exhibited the highest invasive characteristics [31] (Supplementary Fig. 1F and Supplementary Table S4). These findings indicate that the cells within S1 possess the highest stem-like properties among all tumor cells.

We sought to identify the key molecule in the stem-like subtype. It is well-established that maintaining stemness requires the activation of multiple signaling pathways. We found six molecules highly enriched in the stem-like subtype and stem-related pathway (Notch, Wnt, and Hippo pathways) (Fig. 1F). Notably, KLF7 is highly expressed across various tumors, with particularly high expression in head and neck squamous cell carcinoma (Supplementary Fig. 2A). It is overexpressed in OSCC (Fig. 1G, H) and is associated with poor prognosis (Fig. 1I), whereas the other five genes analyzed demonstrated significantly less prognostic relevance than KLF7 (Supplementary Fig. 2B). Furthermore, recent research has shown that KLF7 can re-establish the hematopoietic stem cell niche, suggesting that KLF7 may be a key molecule for maintaining stemness in OSCC [32]. Additionally, immunofluorescence staining of human OSCC tissue revealed a consistent distribution of KLF7, CD44, and CD133 (CSC biomarker, [33, 34]) (Fig. 1J). These findings suggest that elevated KLF7 expression is linked to the stemness properties of OSCC.

#### KLF7 regulates OSCC stemness and migration

**Fig. 2:**
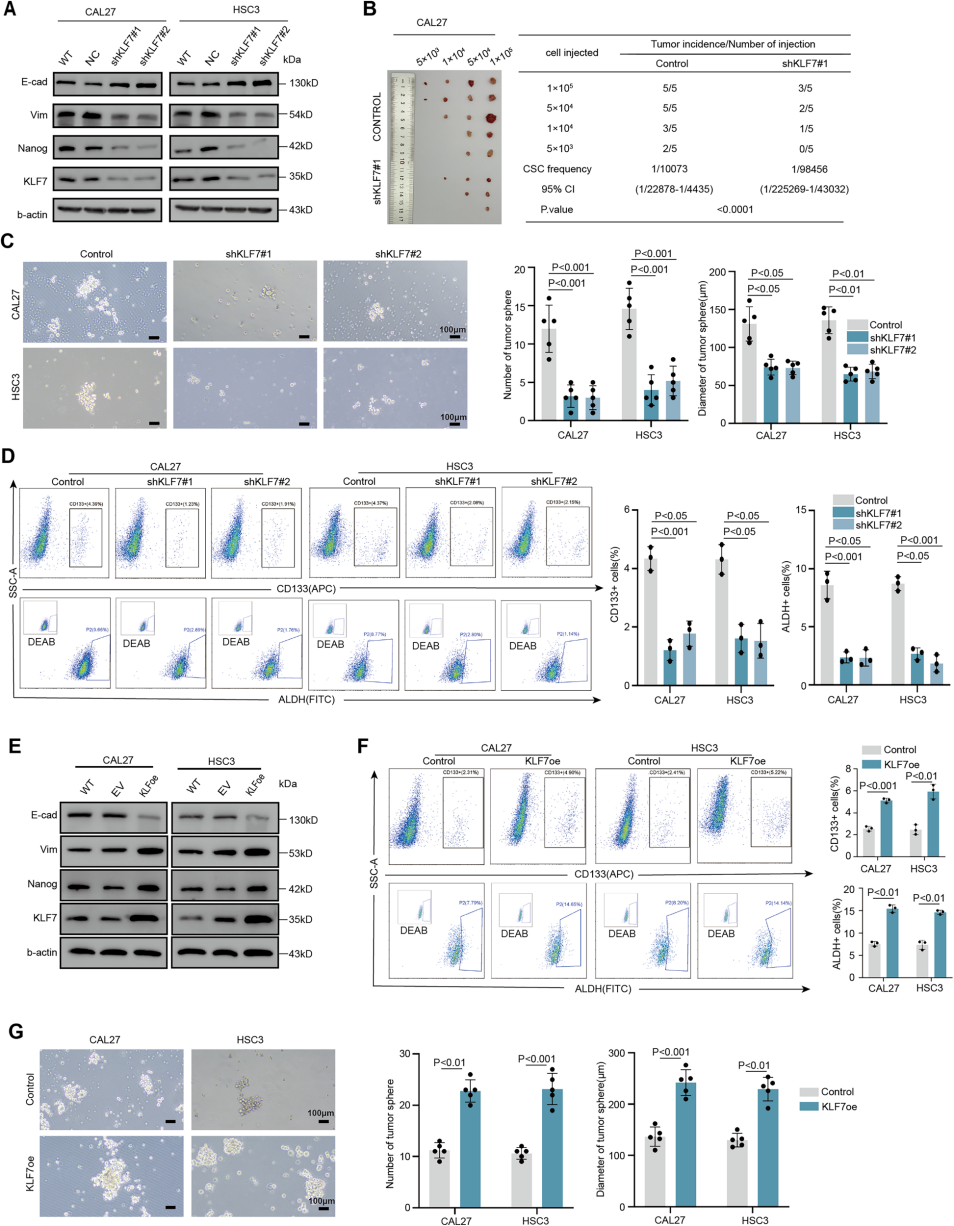
Knockdown KLF7 inhibits the stemness and migration of OSCC. **A** Immunoblot assay of KLF7, NANOG, VIM, and e-cad protein levels in CAL27 and HSC3 cells after stable silencing KLF7. WT wild type, NC Negative control, VIM Vimentin, E-cad E-cadherin. **B** In vivo limiting dilution assays were performed using control cells and KLF7-silenced CAL27 cells, and the frequency of allograft formation at each injected cell dose was determined and is presented (*n* = 5 per group). The data were analyzed using ELDA software. **C** Diameter and number of spheres formed by CAL27 cells and HSC3 cells after stable silencing KLF7; data are presented as mean ± SD from five independent experiments, statistical significance was determined using Student’s *t*-test. Scale bar, 100 μm. **D** The proportion of CD133+ and ALDH+ cells in CAL27 cells and HSC3 cells after stable silencing KLF7; data are presented as mean ± SD from three independent experiments, statistical significance was determined using Student’s *t*-test. **E** Immunoblot assay of KLF7, Nanog, Vim, and E-cad protein levels in CAL27 and HSC3 cells after stable overexpression of KLF7. EV empty vector, oe overexpress. **F** The proportion of CD133+ and ALDH+ cells in CAL27 cells and HSC3 cells after stable overexpression of KLF7; data are presented as mean ± SD from three independent experiments, statistical significance was determined using Student’s *t*-test. **G** Diameter and number of spheres formed by CAL27 cells and HSC3 cells after stable silencing KLF7; data are presented as mean ± SD from five independent experiments, statistical significance was determined using Student’s *t*-test. Scale bar, 100 μm.

To further confirm whether KLF7 regulates stemness in OSCC, we established KLF7-knockdown OSCC cell lines (CAL27 and HSC3), KLF7 knockdown significantly reduced the expression of NANOG, a key transcription factor for maintaining stemness (Fig. 2A). Limiting dilution assays, conducted both in vitro and in vivo, revealed a marked decrease in CSC frequency following KLF7 knockdown (Fig. 2B and Supplementary Fig. 3A). Tumorsphere formation assays showed that KLF7 knockdown significantly reduced both the size and number of tumorspheres, indicating impaired self-renewal capacity (Fig. 2C). Furthermore, flow cytometry analysis revealed that KLF7 knockdown led to a reduction in the proportion of CD133+ cells and impaired aldehyde dehydrogenase (ALDH) activity (Fig. 2D and Supplementary Fig. 4). Conversely, overexpression of KLF7 resulted in increased NANOG expression, enhanced tumor sphere formation, and elevated proportions of CD133+ cells and ALDH activity (Fig. 2E–G). We further examined the impact of KLF7 expression on the proliferative capacity of CAL27 and HSC3 cells and found that KLF7 knockdown did not affect cell proliferation (Supplementary Fig. 3D, E). Collectively, these findings demonstrate that KLF7 plays a critical role in regulating the stemness of OSCC.

Given the reported overlap between epithelial-mesenchymal transition (EMT) and CSC characteristics [35], we analyzed the relationships between KLF7, migration, and EMT. KLF7 knockdown was associated with reduced wound healing rates, fewer migrating cells, decreased vimentin expression, and increased E-cadherin expression (Fig. 2A and Supplementary Fig. 3B, C). Conversely, KLF7-overexpressing CAL27 and HSC3 cell lines enhanced wound healing rates and more migrating cells (Supplementary Fig. 3F, G). Our results suggest that KLF7 plays a crucial role in regulating both OSCC stemness and migration.

#### The stemness-related gene ITGA2 is downstream of KLF7

**Fig. 3:**
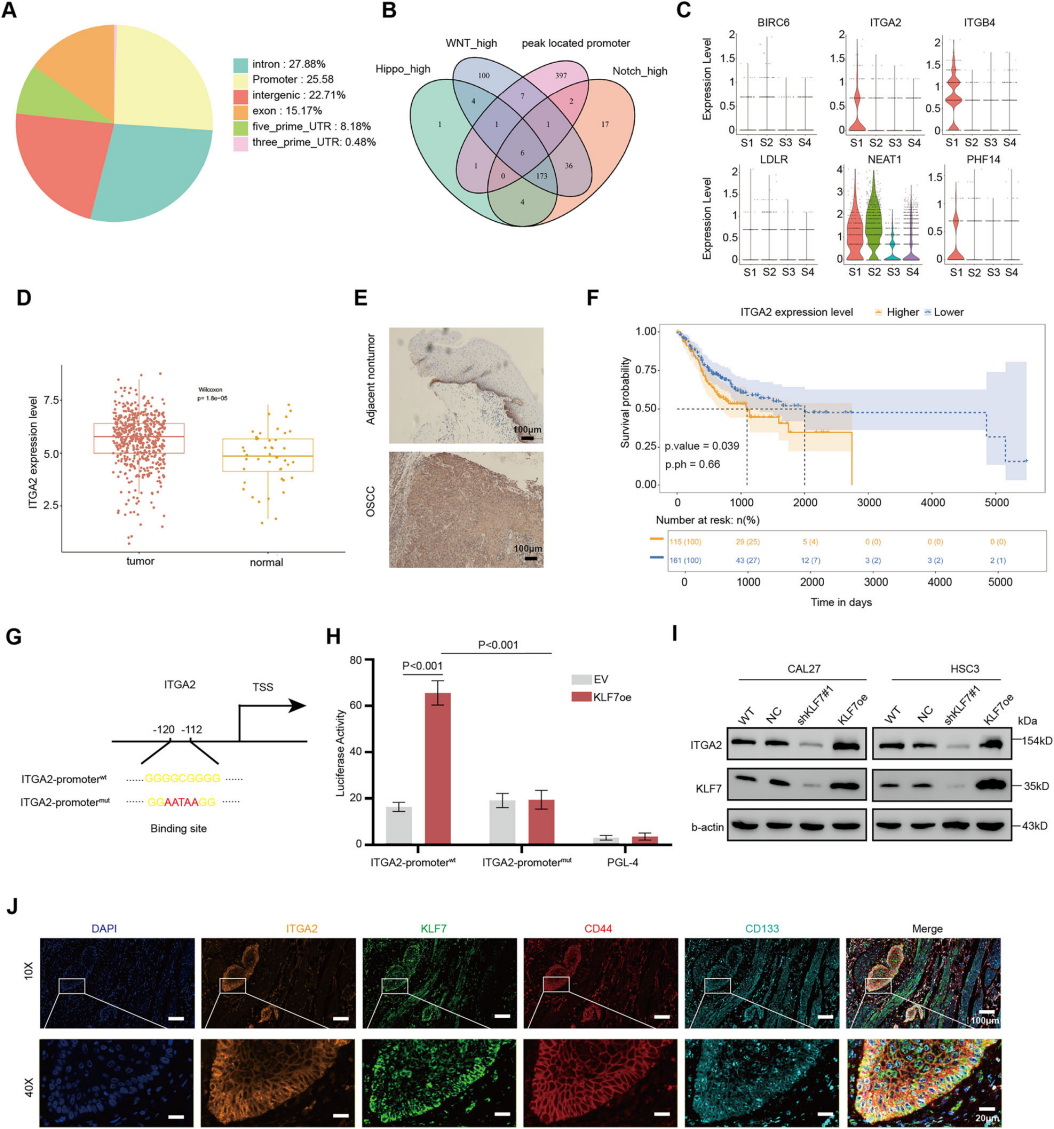
The stem-related ITGA2 is downstream gene of KLF7. **A** The distribution of BATF ChIP-seq peaks. **B** The overlap genes are highly enriched in stem-like subtype, stem-related pathways, and peaks located in the promoter region. **C** Vlnplot of overlap genes expression in OSCC subtype. **D** ITGA2 expression in OSCC and normal samples. **E** Representative immunohistochemistry image showing the expression of ITGA2 in human OSCC sample. Scale bar, 100 μm. **F** Kaplan–Meier curve of ITGA2 in OSCC TCGA dataset. **G** The promoter region of ITGA2 and the binding site are indicated. mut mutant. **H** Luciferase activities of pGP4.19-ITGA2 promoter, mutant constructs, pRL-TK vector, when co-transfected with KLF7+ vector or control. **I** Immunoblot assay of KLF7 and ITGA2 protein levels in CAL27 and HSC3 cells after stable knockdown and overexpressing KLF7. **J** Representative immunofluorescent image showing the distribution of KLF7, ITGA2, CD44, CD133, and DAPI in human OSCC tissue. Scale bar, 100 μm (top); Scale bar, 20 μm (bottom).

KLF7 is a transcription factor capable of activating downstream genes, but it poses challenges for direct drug targeting [36]. To identify downstream target genes with therapeutic potential, we conducted chromatin immunoprecipitation sequencing (ChIP-seq) analysis, focusing on proteins that may be suitable for drug development. After processing the data, we identified 1900 peaks with *q*-values < 0.05 (Supplementary Table S5). Next, we screened for peaks located in the promoter regions of protein-coding genes (Fig. 3A). A total of 179 genes were enriched in the stem-like subset and stemness-related pathways, with six genes identified in ChIP-seq showing peaks located in promoter regions (Fig. 3B). Notably, ITGA2 was specifically enriched in the stem-like subset (Fig. 3C). Furthermore, ITGA2 is highly expressed in OSCC and is associated with poor prognosis, while ITGB4 and PHF14 have no significant effect on OSCC prognosis (Fig. 3D–F and Supplementary Fig. 5A). Our results suggest that ITGA2 may be the downstream gene to maintain stemness in OSCC.

To further confirm the direct regulation of ITGA2 by KLF7, we performed luciferase reporter assays to assess transcriptional activity. Based on the peak location in the ChIP-seq data and predictions from the JSAPAR database, we identified the KLF7 binding site at the −120/−112 region of the ITGA2 promoter (Fig. 3G and Supplementary Fig. 5B). We found that luciferase activity of the pGL4.19 vector, which contains ITGA2 promoter fragments, was significantly increased following transfection of HEK293T cells with the pCMV-KLF7+ vector. Conversely, no such transactivation was observed when cells were transfected with a mutant ITGA2 promoter vector (Fig. 3H). Western blot analysis confirmed that ITGA2 expression is regulated by KLF7, with ITGA2 levels decreasing upon KLF7 knockdown and increasing with KLF7 overexpression (Fig. 3I). Immunofluorescence staining of OSCC samples revealed a consistent distribution of ITGA2, KLF7, CD44, and CD133 further supporting the link between KLF7, ITGA2, and stemness (Fig. 3J). This funding suggests that the stem-related gene ITGA2 is downstream of KLF7.

#### ITGA2 regulates OSCC stemness and migration

**Fig. 4:**
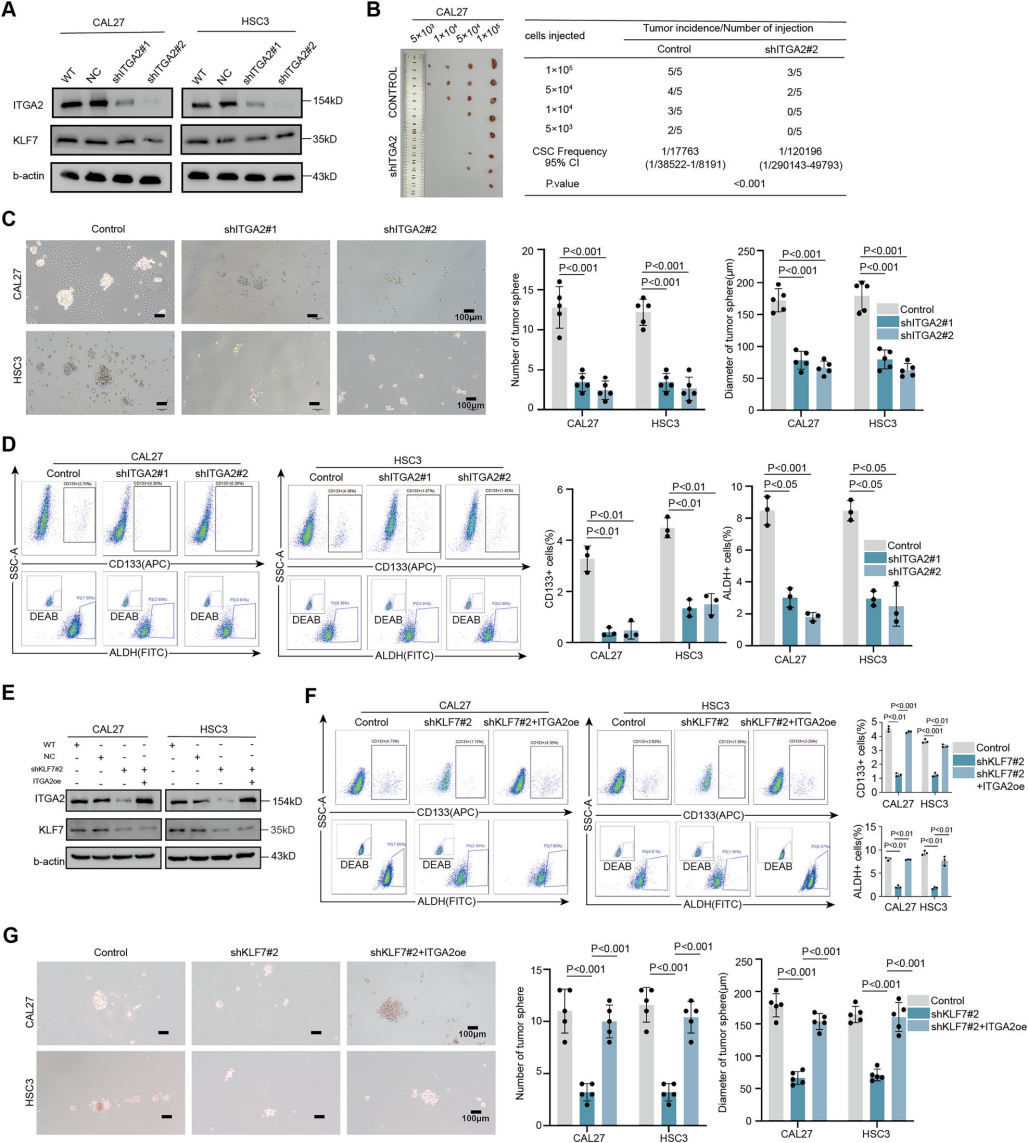
Knockdown ITGA2 inhibits the stemness of OSCC. **A** Immunoblot assay of KLF7 and ITGA2 protein levels in CAL27 and HSC3 cells after stable silencing ITGA2. WT wild type, NC Negative control. **B** In vivo limiting dilution assays were performed using control cells and ITGA2-silenced CAL27 cells, and the frequency of allograft formation at each injected cell dose was determined and is presented (*n* = 5 per group). The data were analyzed using ELDA software. CI Confidence Interval. **C** Diameter and number of spheres formed by CAL27 cells and HSC3 cells after stable silencing ITGA2; data are presented as mean ± SD from five independent experiments, and statistical significance was determined using Student’s *t*-test. Scale bar, 100 μm. **D** The proportion of CD133+ and ALDH+ cells in CAL27 cells and HSC3 cells after stable silencing ITGA2; data are presented as mean ± SD from three independent experiments, statistical significance was determined using Student’s *t*-test. **E** Immunoblot assay of KLF7 and ITGA2 protein levels after stable silencing ITGA2 and silencing KLF7 followed by stable overexpression of ITGA2. **F** The proportion of CD133+ and ALDH+ cells in CAL27 cells and HSC3 cells after stable silencing KLF7 and silencing KLF7 followed by stable overexpression of ITGA2; data are presented as mean ± SD from three independent experiments, statistical significance was determined using Student’s *t*-test. **G** Diameter and number of spheres formed by CAL27 cells and HSC3 cells after stable silencing KLF7 and silencing KLF7 followed by stable overexpression of ITGA2; data are presented as mean ± SD from five independent experiments, and statistical significance was determined using Student’s *t*-test. Scale bar, 100 μm.

To investigate whether ITGA2 regulates OSCC stemness, we established ITGA2-knockdown CAL27 and HSC3 cell lines (Fig. 4A). Limiting dilution assays, conducted both in vitro and in vivo, revealed a significant reduction in CSC frequency upon ITGA2 knockdown (Fig. 4B and Supplementary Fig. 6A). Tumorsphere formation assays showed a marked decrease in both the size and number of tumorspheres in ITGA2-knockdown cells, indicating impaired self-renewal capacity of CSC (Fig. 4C). Flow cytometry analysis further demonstrated that ITGA2 knockdown reduced the proportion of CD133+ cells and diminished ALDH activity (Fig. 4D). Consistent with the regulatory role of KLF7, ITGA2 knockdown also attenuated wound closure rates and decreased the number of migrating cells (Supplementary Fig. 6B, C). Notably, proliferation assays confirmed that ITGA2 knockdown had no impact on the proliferative capacity of CAL27 and HSC3 cells (Supplementary Fig. 6D, E). These findings collectively indicate that ITGA2 is critically involved in regulating OSCC stemness and migration.

Next, we overexpressed ITGA2 in KLF7-knockdown cell lines. ITGA2 overexpression reversed the reduction in tumorsphere size and number, CD133+ cell proportion, ALDH activity, wound healing, and migrating cell numbers caused by KLF7 knockdown (Fig. 4E–G and Supplementary Fig. 6F, G). These findings highlight the critical role of the KLF7/ITGA2 axis in regulating stemness and migration in OSCC.

#### ITGA2 binds to type I collagen to drive YAP nuclear translocation and activate the stemness pathway

**Fig. 5:**
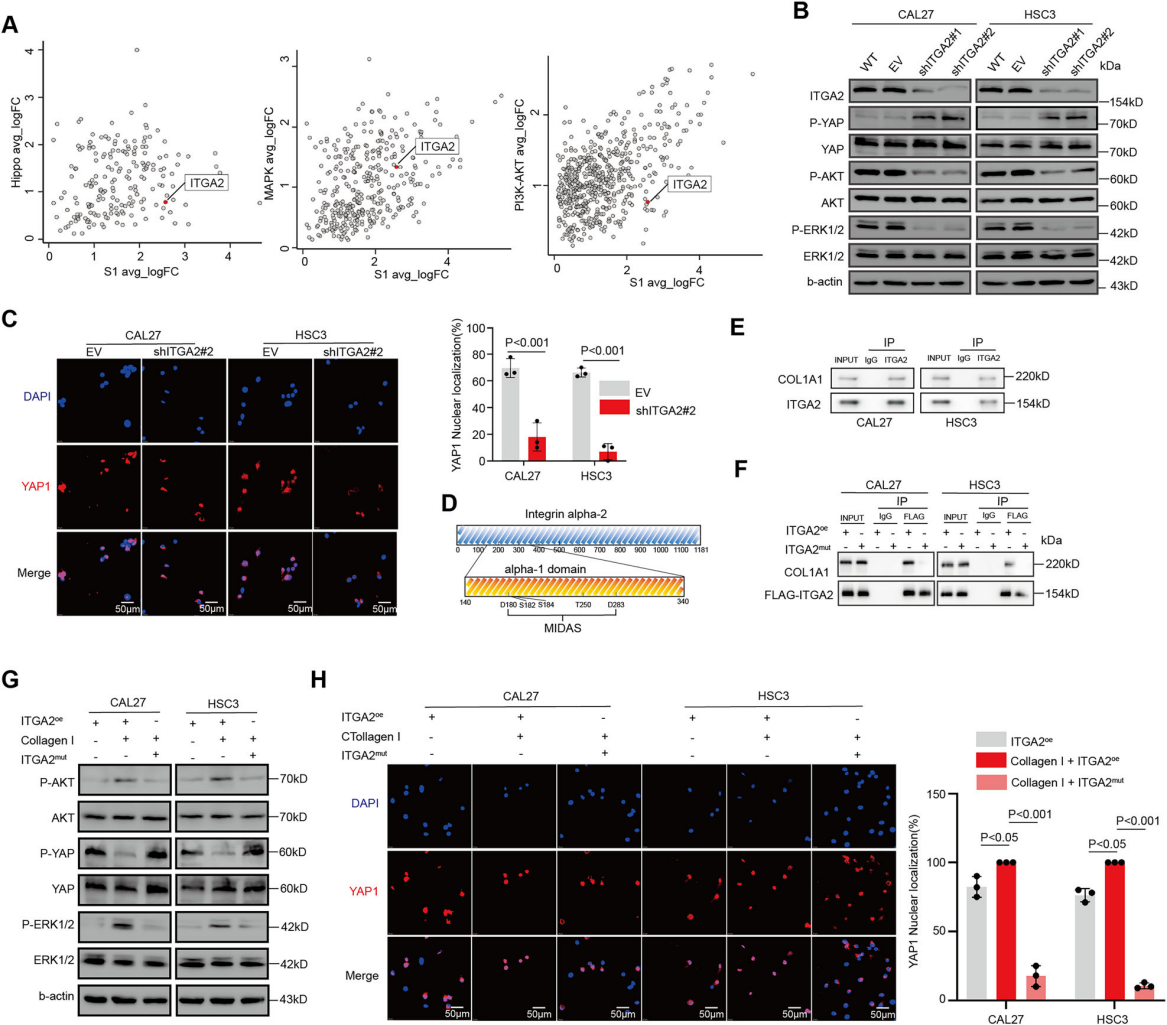
ITGA2 binds to type I collagen to drive YAP nuclear translocation and activate the stemness pathway. **A** The log2-transformed fold change (FC) in RNA levels between the stem-like cluster with the rest of the clusters and the stem-related pathway with the rest of the pathway. **B** Immunoblot assay of ITGA2, phosphor-ERK1/2, total-ERK1/2, phosphor-AKT, total-AKT, phosphor-YAP1, total-YAP1 protein levels in CAL27 and HSC3 cells after stable silencing ITGA2. p phosphor. **C** Confocal detected the nuclear localization of YAP1 in CAL27 and HSC3 cells after transduced with shITGA2#2; data are presented as mean ± SD from three independent experiments, and statistical significance was determined using Student’s *t*-test. Scalebar, 50 μm. **D** The sequence and domain in ITGA2 protein. **E** Co-immunoprecipitation suggested direct molecular interaction between ITGA2 and COL1A1 proteins in CAL27 and HSC3 cells. **F** Co-IP analysis in CAL27 and HSC3 cells transduced with ITGA2^OE^ and ITGA2^mut^ plasmid. **G** Immunoblot assay of phosphor-ERK1/2, total-ERK1/2, phosphor-AKT, total-AKT, phosphor-YAP1, total-YAP1 protein levels in CAL27 and HSC3 cells after transduced with ITGA2^OE^ and ITGA2^mut^ plasmid. **H** Confocal detected the nuclear localization of YAP1 in CAL27 and HSC3 cells after transduced with ITGA2^OE^ and ITGA2^mut^ plasmid; data are presented as mean ± SD from three independent experiments, and statistical significance was determined using Student’s *t*-test. Scalebar, 50 μm.

Although we have established that ITGA2 regulates OSCC stemness, the precise mechanisms remain unclear. As a member of the integrin family, ITGA2 functions in responding to mechanical stimuli from the extracellular matrix. The Hippo pathway, a downstream effector of mechanical signals, is extensively linked to and interacts with integrins [37–39]. In addition to classical developmental signaling pathways such as Notch, Hippo, and WNT, the AKT and MAPK signaling pathways are also critical for tumor initiation. These pathways interact with developmental signaling pathways and influence tumor stemness [40–43]. Based on single-cell analysis of differentially expressed genes (DEGs) in these pathways, we found that ITGA2 is potentially associated with the activation of the Hippo, MAPK, and AKT pathways (Fig. 5A). Knockdown of ITGA2 decreased phosphorylation levels of AKT and ERK1/2, increased YAP1 phosphorylation, and led to YAP1 nuclear export (Fig. 5B, C). ITGA2 contains an additional domain (“A” or “I”) within the head region, with a conserved metal ion-dependent adhesion site (MIDAS) motif, which is crucial for ligand binding at the C-terminal of the type I domain (Fig. 5D) [44–46]. Mutagenesis studies show that the MIDAS motif and exposed side chains on the surrounding surface are required for ligand binding [44, 47–49].

ITGA2 mediates interactions between cells and the extracellular matrix (ECM), with type I collagen as the primary ECM component [50]. To investigate whether ITGA2 interacts with type I collagen and activates stemness-related pathways, we constructed and transfected OSCC cells with ITGA2^oe^ or an ITGA2^mut^(MIDAS mutant). IP results confirmed an interaction between ITGA2 and COL1A1, which was abolished in ITGA2-mutant (Fig. 5E, F). Upon the addition of type I collagen to the culture, the phosphorylation of AKT and ERK1/2 increased, and the phosphorylation level of YAP1 decreased, leading to YAP1 nuclear translocation. while phosphorylation of AKT and ERK1/2 decreased, YAP1 increased and YAP1 nuclear export in ITGA2-mutant (Fig. 5G, H). These findings suggest that the MIDAS domain of ITGA2 structurally interacts with type I collagen, thereby activating the PI3K/AKT, MAPK, and HIPPO/YAP1 pathways to maintain the stemness of OSCC.

#### TC-I 15 inhibits OSCC and augments the chemotherapy responsiveness of OSCC

**Fig. 6:**
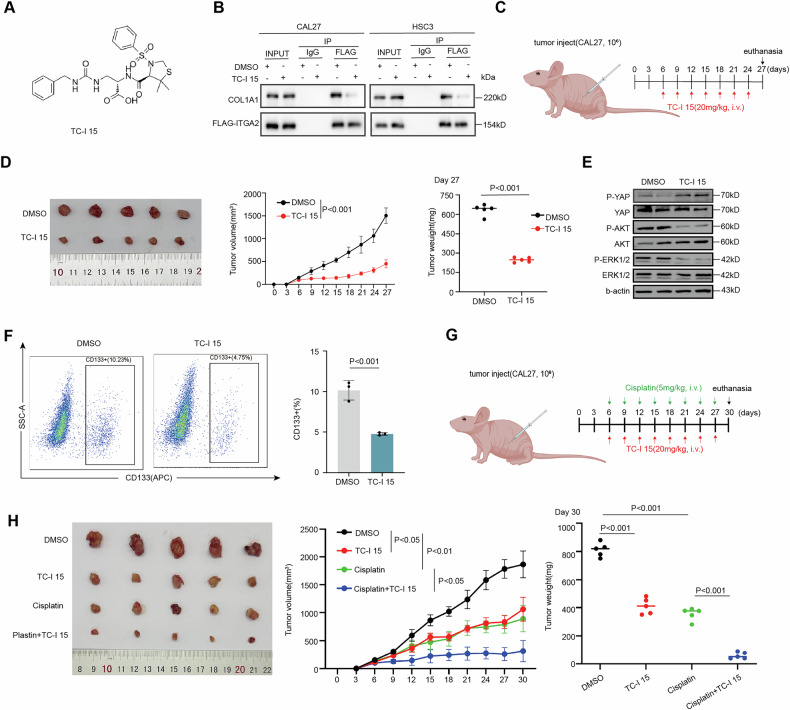
TC-I 15 inhibits OSCC and augments chemotherapy responsiveness of OSCC. **A** The molecular formula of TC-I 15. **B** Co-IP analysis in CAL27 and HSC3 cells treated with DMSO and TC-I 15. **C** CAL27 cells were intracardially injected into mice. Six days later, TC-I 15 was injected (20 mg/kg) into mice via vein every third day. **D** Representative images showing the xenograft model in CA27 cells treated with DMSO or TC-I 15 (*n* = 5 per group) and the growth of tumor grafts was shown; data are presented as mean ± SD, statistical significance was determined using Student’s *t*-test. DMSO Dimethylsulfoxide. **E** Immunoblot assay of phosphor-ERK1/2, total-ERK1/2, phosphor-AKT, total-AKT, phosphor-YAP1, and total-YAP1 protein levels in xenograft tissue. p phosphor. **F** Representative FACS plots and quantification of CD133 + cells in the xenograft tissues; data are presented as mean ± SD from three independent experiments, statistical significance was determined using Student’s *t*-test. **G** CAL27 cells were intracardially injected into mice. Six days later, TC-I 15 (20 mg/kg) and Cisplatin (5 mg/kg) were injected into nude mice via vein every third day. **H** Representative images showing the xenograft model in CA27 cells treated with DMSO or TC-I 15 (*n* = 5 per group) and the growth of tumor grafts were shown; data are presented as mean ± SD, and statistical significance was determined using Student’s *t*-test.

TC-I 15 is an allosteric collagen-binding integrin α2β1 inhibitor, with half-maximal inhibitory concentration (IC50) values of 26.8 μM and 0.4 μM for binding to GFOGER and GLOGEN, respectively (Fig. 6A). During the binding of type I collagen to integrin α2β1, the I domain of integrin α2 coordinates a divalent cation, Mg2+, at its MIDAS [46, 51]. The crystal structure of the interaction between integrin α2β1 and the triple-helical motif (GFOGER) of type I collagen has been resolved. The GFOGER motif and the integrin I domain form a complex through coordination with Mg2+, a mechanism that underscores the critical role of the carboxylate side chain of glutamic acid (E) in the GxOGEx sequence (including GLOGEN) for integrin-mediated binding [46]. Consequently, We selected TC-I 15 for both in vivo and in vitro experiments to evaluate the feasibility of targeting ITGA2 for OSCC treatment. Immunoprecipitation assays showed that TC-I 15 interferes with the binding between ITGA2 and type I collagen (Fig. 6B). In an in vivo experiment, CAL27 cells were xenografted into nude mice, and from day 6 post-inoculation, TC-I 15 were intravenously injected every 3 days, with tumor volume measured over time (Fig. 6C). The results showed that the TC-I 15 significantly reduced tumor size, growth rate, and tumor weight (Fig. 6D). Moreover, TC-I 15 decreased phosphorylation levels of AKT and ERK1/2, increased YAP1 phosphorylation, and decreased CD133+ cells determined by flow cytometry (Fig. 6E, F).

Given the critical role of CSCs in tumor drug resistance, we further investigated whether TC-I 15 could enhance the sensitivity of OSCC to chemotherapy. CAL27 cells were xenografted into nude mice, and starting from day 6 post-inoculation, cisplatin and TC-I 15 were administered intravenously every 3 days. Tumor volume was monitored and measured over time (Fig. 6G). The results showed that the combination of TC-I 15 and cisplatin significantly reduced tumor size, growth rate, and tumor weight compared to cisplatin alone (Fig. 6H). These findings suggest that TC-I 15 inhibits OSCC and enhances the sensitivity of OSCC to chemotherapy, highlighting the potential of targeting ITGA2 to improve clinical anti-tumor efficacy.

## Discussion

Our study revealed that KLF7 modulates stemness properties in OSCC. The KLF family is closely associated with CSCs. Notably, KLF4 plays a crucial role in maintaining CSC characteristics in liver and breast cancers [52, 53]. Additionally, KLF11 has been identified as a negative regulator in sarcoma CSCs, where its epigenetic silencing leads to prolonged YAP activation and poor prognosis [54]. KLF7 can also enhance cancer cell migration, and invasion across various cancer types [55, 56]. Although direct evidence linking KLF7 to CSC regulation is lacking, its ability to modulate genes involved in the EMT suggests a potential impact on CSC properties [57]. Our study addresses this gap by establishing the correlation between KLF7 and CSC traits in OSCC, broadening the understanding of the KLF family’s diverse functions.

Cancer stemness is strongly associated with metastasis, treatment resistance, and immune evasion. Therefore, developing therapeutic strategies that target cancer stemness could substantially enhance treatment outcomes [35]. Although we have uncovered the link between KLF7 and stemness, as a transcription factor, KLF7 is considered undruggable due to its structural and functional complexities [58]. A more viable strategy is to focus on downstream molecules that play crucial roles in the KLF7-mediated regulation of stemness and are more amenable to therapeutic targeting. Using ChIP-seq and functional assays, we identified ITGA2 as a key downstream regulator of stemness influenced by KLF7.

ITGA2, a member of the integrin family, is a cell surface protein that acts as a crucial node, transmitting signals from the ECM to the intracellular environment and triggering downstream pathways [59]. Recent evidence suggests that CSCs represent a plastic cellular state, dynamically shaped by the interaction between CSCs and their niche [60]. Various components within the tumor niche, such as cancer-associated fibroblasts and immune cells, can influence stemness through mechanisms involving cytokines or metabolites [61, 62]. Considering that differentiated tumor cells can acquire stem-like properties within a CSC-supportive environment, directly eliminating CSCs may not be a practical approach. Consequently, there has been growing research interest in targeting the CSC niche or disrupting the interactions between CSCs and their surrounding environment. The ECM is a key component of the tumor niche, and ECM–tumor or ECM–immune cell interactions play a crucial role in promoting CSC properties [62, 63]. Therefore, targeting the ECM and its associated signaling pathways presents a promising strategy to inhibit cancer stemness.

Our findings demonstrate that ITGA2 is enriched in cancer cells with high stemness and regulates the stemness of OSCC. Similar to our findings, ITGA2 has been shown to be enriched in glioma stem cells and co-expressed with the stem cell marker SOX2 [64]. Furthermore, elevated ITGA2 expression has been observed in glioblastoma CSCs compared to their differentiated counterparts [65]. Our research further suggests that upon binding to type I collagen, ITGA2 activates several crucial pathways involved in stemness, proliferation, survival, and angiogenesis. Based on these findings, we propose that ITGA2 plays a crucial role in mediating the interactions within the CSC niche. By overexpressing ITGA2, CSCs can receive more signals that reinforce their stemness, positioning ITGA2 as a promising therapeutic target.

Additionally, as a membrane protein, ITGA2 is highly amenable to drug targeting. Several agents targeting ITGA2, including blocking antibodies and small-molecule inhibitors, are available [66, 67]. Our in vitro and in vivo experiments suggest that ITGA2-specific inhibitors can suppress the stemness of OSCCs and increase their sensitivity to cytotoxic drugs. We are particularly interested in exploring whether blocking antibodies targeting ITGA2 could elicit similar effects, and further, whether ITGA2 inhibitors could enhance the efficacy of immunotherapy.

Despite the robust evidence presented in this study, several limitations exist. First, most of our experiments were conducted in vitro, and additional in vivo studies are needed to provide more comprehensive evidence. Second, this study used OSCC cell lines, and future studies employing patient-derived xenografts or tumor organoids could improve the generalizability of our findings. In conclusion, we have identified KLF7 as a regulator of CSC properties in OSCC through its enhancement of ITGA2-mediated ECM-supported stemness acquisition. Moreover, we demonstrated the feasibility of targeting ITGA2 as a potential therapeutic approach.

## Method

### Single-cell RNA-seq data processing

Single-cell transcriptome sequencing data from OSCC samples were obtained from GSE234933 and GSE82227. The Cell Ranger pipeline (v7.1.0) was used for processing raw scRNA-seq data, including demultiplexing, barcode handling, alignment, and preliminary clustering. Sequencing reads were mapped, annotated, and quantified against the GRCh38 reference annotation (available at https://cf.10xgenomics.com/supp/cell-exp/refdata-gex-GRCh38-2020-A.tar.gz). The UMI count matrix was filtered using the Seurat [68] *R* package (v5.0.3), applying a criterion based on the mean value ± 2 times the median absolute deviation of UMI/gene counts per cell, assuming a Gaussian distribution. Following a visual inspection, additional low-quality cells, particularly those with >25% mitochondrial gene counts, were excluded. The DoubletFinder package [69] (v2.0.2) was used to detect potential doublets. Samples were treated as separate batches, and batch effects were addressed using the Harmony algorithm [70]. After applying these quality control measures, 55,880 high-quality single cells remained for further analysis. The normalized expression profiles of all samples were combined using the Merge() function in *R*. Library size normalization, log-transformation, and selection of the top 3000 variable genes were performed using the SCTransform() function in Seurat.

Principal components (PCs) were derived from the expression data of these 3000 genes. Cell clustering was carried out using the FindNeighbors() and FindClusters() functions in Seurat. For visualization, the RunUMAP() and DimPlot() functions were employed to generate 2D Uniform Manifold Approximation and Projection (UMAP) plots. Marker genes for each cluster were determined using the FindAllMarker() function in Seurat, which identified positive markers for a cluster compared to all other clusters.

CNV analysis was performed using the HoneyBADGER [71] package (ver. 0.1.14). To detect DEGs, we used the FindMarkers() function in the Seurat package, setting the test.use parameter to “presto”. For assessing pathway activities at the single-cell level, we implemented GSVA [72] with the default settings provided in the GSVA package (v1.50.1). The gene set file was loaded using the GSEABase package (v1.64.0) and the minder package (v7.5.1). This file was obtained from both the Kyoto Encyclopedia of Genes and Genomes (KEGG) database (available at https://www.kegg.jp/) and the msigdbr resource (available at https://www.gsea-msigdb.org/gsea/msigdb). Cell trajectory inference was performed using the Monocle 3 (ver. 1.3.6) [73] packages. The CytoTRACE 2 [74] tool was used to compute potency scores and assess the state of cell differentiation. AUCell [75] was used to measure stemness and invasiveness activity.

### Patients and sample collection

The OSCC tissue microarrays containing 42 cases of specimens were used for KLF7, ITGA2, CD44, and CD133 staining. The patient studies were conducted according to the Declaration of Helsinki, and the use of these specimens and data for research purposes was approved by the Ethics Committee of Zhejiang University, School of Medicine, First Affiliated Hospital.

### TCGA analysis

RNA sequencing and corresponding clinical data for head and neck squamous cell carcinoma were downloaded from The Cancer Genome Atlas (TCGA) database (https://www.cancer.gov/ccg/research/genome-sequencing/tcga). We extracted RNA-seq data from 334 primary tumor specimens, mainly located in the oral cavity, and 44 normal specimens. Additionally, RNA-seq data and clinical information from 33 tumor types were obtained from the TCGA database using Xena (https://xenabrowser.net) for pan-cancer analysis. Wilcoxon tests were performed to analyze significant differences. Kaplan–Meier survival curves were used to assess survival differences between groups, with the log-rank test used to determine statistical significance.

### Cell culture

OSCC cell lines (CAL27 and HSC3) and HEK293T cells were obtained from the Oral Laboratory of Zhejiang University. All cell lines were cultured in Dulbecco’s modified Eagle medium (DMEM) supplemented with 10% fetal bovine serum and 1% penicillin/streptomycin and maintained at 37 °C in 5% CO_2_. Cells were passaged at 70–90% confluency, and mycoplasma contamination was regularly checked using a detection kit.

### Type I collagen

To investigate the interaction between ITGA2 and type I collagen, 5 × 10^6^ cells were cultured in 6-well plates (Corning) pre-coated with human type I collagen (C7774, Sigma-Aldrich; 5 μg/cm^2^).

### Samples and animals

Formalin-fixed, paraffin-embedded patient samples were retrospectively obtained from the First Affiliated Hospital of Zhejiang University. NOD/SCID nude rats were purchased from the Second Affiliated Hospital of Zhejiang University. The study adhered to the Declaration of Helsinki, and the study protocol was approved by the Ethics Committee of both the First and Second Affiliated Hospitals of Zhejiang University.

### Plasmids and lentiviral transfection

The KLF7 overexpression and knockdown lentiviral vectors were obtained from Vigene (Shandong, China), and the ITGA2 lentiviral vector was sourced from GenePharma (Shanghai, China). The promoters of wild-type and mutant ITGA2 were cloned into the pGL4.19 vector. The ITGA2 plasmid was synthesized in our laboratory: full-length human ITGA2 was amplified from HEK293T cDNA and cloned into the Xho I and EcoR I restriction sites of the pcDNA3.1-Flag vector using the ClonExpress MultiS One Step Cloning Kit (#C113-02; Vazyme). ITGA2 mutants were generated by site-directed mutagenesis using the Mut Express MultiS Fast Mutagenesis Kit (#C215-01; Vazyme) according to the manufacturer’s instructions. To establish lentiviral transfected cell lines, lentiviruses were added to CAL27 and HSC3 cells, and 24 h later, the medium was replaced with a medium containing 2 µg/mL puromycin. Plasmid transfections were carried out using Lipofectamine 3000 (Invitrogen, Carlsbad, CA, USA) according to the manufacturer’s instructions. The primer sequences are presented in the Supplementary Table S6.

### Scratch wound assay

For scratch wound assays, cells were seeded into 6-well plates and grown to confluence. A 200-µL pipette tip was used to create a scratch wound, followed by two washes with phosphate-buffered saline to remove detached cells. Serum-free medium was added, and wound closure was monitored under the microscope every 3 h. ImageJ software was used to analyze the wound closure area and calculate the wound healing rate.

### Transwell migration assay

Serum-free medium was used to prepare HSC3 and CAL27 cell suspension, 200 µL of cell suspension was added to the upper chamber of the Transwell (3403, Corning) while 800 µL of complete culture medium was added to the lower chamber. After incubation for 48 h, 4% paraformaldehyde-fixed the cells that passed through the cell chamber filter attached to the surface and stained with 0.4% crystal violet. ImageJ software was used to measure the transwell migration area.

### Cell proliferation and colony formation assays

CCK-8 assays were carried out to determine the function of KLF7 and ITGA2 on cell proliferation. In brief, cells were seeded in each well of 96 well plates (5 × 10^3^ cells/well), and CCK-8 solution was added 24, 48, and 72 h after placing. Cells were incubated at 37 °C for 1 h after 10 μl CCK-8 solution was added. For the colony formation assay, 500 cells per well were seeded into 24-well plates (Corning) and cultured at 37 °C for 14 days. Visible colonies were washed twice with phosphate-buffered saline (PBS), fixed with 4% paraformaldehyde, and stained with crystal violet. Colony numbers were quantified using ImageJ software.

### Immunohistochemistry and immunofluorescence

All paraffin-embedded tissues were sectioned into 4-μm slices. After deparaffinization, rehydration, antigen retrieval, and blocking, the sections were incubated overnight with primary antibodies. After incubation at 37 °C for 50 min with Goat Anti-Rabbit IgG H&L (HRP) antibodies (ab205718, Abcam, 1:2000), the sections were stained with 3,3′-diaminobenzidine (DAB) and counterstained with hematoxylin. For multiplex immunofluorescence staining of human oscc tissue samples, we used the Opal Polaris 7-Color Manual IHC Detection Kit (NEL861001KT; Akoya Biosciences) following the manufacturer’s instructions. Briefly, after routine blocking and incubation with primary and secondary antibodies, tyramide signal amplification staining was performed. Subsequently, the second, third, and fourth antibodies were incubated, followed by DAPI staining. The antibodies used in this study included KLF7 (ab197690, Abcam, 1:200), ITGA2 (A19068, ABclonal,1:400), CD133 (18470-1-AP, Proteintech, 1: 200), and CD44 (A21919, Abclonal, 1:800). For cellular immunofluorescence, cells were plated on coverslips and fixed with paraformaldehyde upon reaching 30% confluence, followed by permeabilization. After washing and blocking, the primary antibody against YAP1 (66900-1-Ig, Proteintech, 1:400) was incubated overnight. The slides were then incubated with the Goat Anti-Mouse IgG H&L (Alexa Fluor® 647) (ab150115, Abcam, 1:200) and counterstained with DAPI.

### Immunoblotting

Cells were lysed in RIPA buffer containing protease inhibitors. After scraping, lysates were centrifuged, and supernatants were collected. Protein concentrations were measured using a BCA kit. After Sodium dodecyl sulfate-polyacrylamide gel electrophoresis (SDS-PAGE) and membrane transfer, blocking was performed, and primary antibodies were incubated overnight at 4 °C. The primary antibodies used in this study included anti-KLF7(ab197690, Abcam, 1:1000), anti-ITGA2(A19068, ABclonal, 1:1000), anti-E-cadherin (ab40772, Abcam, 1:5000), anti-vimentin (A19607, Abclonal, 1:1000), anti-β-actin (8457S, CST, 1:1000), anti-NANOG (D73G4, CST, 1:1000), anti-AKT (9272S, CST, 1:1000), anti-p-AKT (4060S, CST, 1:1000), anti-ERK1/2 (4695S, CST, 1:1000), anti-p-ERK1/2 (4370S, CST, 1:1000), anti-YAP1 (66900-1-Ig, Proteintech, 1:1000), anti-p-YAP1 (29018-1-AP, Proteintech, 1:1000), and anti-COL1A1 (A25310, Abclonal, 1:1000). The secondary antibodies used in this study: Anti-rabbit IgG, HRP-linked Antibody (7074S, CST, 1:2000), Anti-mouse IgG, HRP-linked Antibody (7076S, CST, 1:2000).

### Immunoprecipitation

For immunoprecipitation, cells or tissues were homogenized in lysis buffer. Protein extracts were incubated with Pierce™ anti-DYKDDDDK Magnetic Agarose (Thermo Fisher Scientific). The binding complexes were washed with buffer, mixed with loading buffer, and subjected to SDS-PAGE. The presence of ITGA2 in the precipitates was detected using a rabbit anti-ITGA2 antibody (A19068, ABclonal, 1:1000), and collagen 1 was detected using an anti-COL1A1 antibody (A25310, Abclonal, 1:1000).

### Sphere formation assay

To assess sphere formation, cells(2000 cells per well) were cultured in ultra-low adhesion 6 plates (Corning) in serum-free DMEM/F12 supplemented with 20 ng/mL epidermal growth factor(PMG8045, Thermo Scientific), 20 ng/mL basic fibroblast growth factor (13256029, Thermo Scientific), and 1× B27 (17504044, Thermo Scientific). The number and diameter of tumorspheres with a diameter >50 μm were quantified after 7–10 days of culture.

### Flow cytometry

For flow cytometry, cells were collected and resuspended at a concentration of 1 × 10^6^ cells per 200 µL of phosphate-buffered saline per tube. Cells were stained with allophycocyanin (APC)-conjugated anti-CD133 antibody (566596, BD Pharmingen, 1:100) on ice for 30 min, washed two or three times, and analyzed. An ALDEFLUOR kit (01700, STEMCELL Technologies) was used to analysis ALDH enzymatic activity, each test tube (500 μl system) was added with 2.5 μl ALDEFLUOR reagent and each Diethylaminoazobenzene (DEAB) tube was added with 2.5 μl ALDEFLUOR reagent as well as 5 μl DEAB reagent and the ALDH+ population was determined according to the manufacturer’s instructions. Data were analyzed using Flowjo software (10.8.1) (BD biosciences, USA).

### Limiting dilution assay

In vivo limiting dilution assays, cells were suspended at various densities (1 × 10^5^, 5 × 10^4^, 1 × 10^4^, and 5 × 10^3^ cells per 100 µL) in serum-free DMEM containing 10% Matrigel and injected into 6–8-week-old female nude mice. Tumor growth was monitored, and after 3 weeks, the mice were sacrificed for sample collection. In vitro limiting dilution assays, Different amounts of Control cells,shKLF7 cells, shITGA2 cells (1000, 500, 100, 50 cells/well) were cultured in ultra-low adhesion 96 plates (Corning) in serum-free DMEM/F12 supplemented with 20 ng/mL epidermal growth factor(PMG8045, Thermo Scientific), 20 ng/mL basic fibroblast growth factor (13256029, Thermo Scientific), and 1× B27 (17504044, Thermo Scientific). The estimated range for tumor sphere-forming cells or tumor-initiating cells were calculated using https://bioinf.wehi.edu.au/software/elda/.

### ChIP-seq analysis

The establishment of MYC-KLF7 + -expressing CAL27 cells and ChIP-seq were performed by Igenebook Biotechnology Co. Ltd. (Wuhan, China). Briefly, 1 × 10^7^ cells were washed and cross-linked with 1% formaldehyde, and then quenched by adding glycine. Cells were lysed, and chromatin was isolated on ice. Chromatin was sonicated to produce soluble, sheared chromatin. Next, 20 µL of chromatin was saved for input DNA, whereas 100 µL of chromatin was used for immunoprecipitation with anti-MYC antibodies (D3N8F, CST). The immunoprecipitated material was bound to protein beads, washed, and eluted from the beads. The eluted material was treated with RNase A followed by proteinase K. The immunoprecipitated DNA was used to construct sequencing libraries, which were sequenced on an NovaSeq 6000 (Illumina, San Diego, CA, USA) using paired-end 150 bp sequencing.

### ChIP-seq data analysis

Trimmomatic (ver. 0.36) was used to filter out low-quality reads [76]. Clean reads were mapped to the human genome using BWA (ver. 0.7.15) [77]. Samtools (ver. 1.3.1) was applied to remove potential PCR duplicates [78]. Peak calling was performed using MACS2 (ver. 2.1.1.20160309) with default parameters (bandwidth, 300 bp; model fold, 5, 50; *q* value, 0.05). Peaks were assigned to genes if their midpoints were closest to the transcription start site of a gene [79]. Motif occurrence within peaks was predicted using HOMER (ver. 3) with a maximum motif length of 12 base pairs [80]. The clusterProfiler [81] (http://www.bioconductor.org/packages/release/bioc/html/clusterProfiler.html) package in *R* software [was employed to perform Gene Ontology (http://geneontology.org/) [82] and KEGG (http://www.genome.jp/kegg/) enrichment analysis [83], with a hypergeometric distribution and a *q* value cutoff of 0.05 [81].

### Luciferase reporter assays

For the luciferase reporter assays, CAL27 cells were seeded onto 24-well plates and transfected with pGP4.19-ITGA2 promoter, mutant constructs, and pRL-TK vector, when co-transfected with KLF7+ vector or control for 24 h. Cells were then harvested and lysed using lysis buffer (Promega Biosciences) at room temperature for 15 min. Luciferase activity was measured using the Dual Luciferase Reporter Assay System Kit (Promega Biosciences) according to the manufacturer’s instructions. Total light production (optical density: 490 nm) was measured using the SpectraMax M3 multimode microplate reader (Molecular Devices) and normalized to Renilla luciferase activity.

### Xenograft

For in vivo experiments, tumor cells were suspended in serum-free DMEM and injected subcutaneously into the axillae of 4–6-week-old nude mice. Tumor growth was measured using calipers every three days and recorded in cubic centimeters. In inhibitor treatment experiments, starting on day six post-inoculation, cisplatin (A821, APExBIO, 5 mg/kg) or TC-I 15 (HY-107588, MCE, 20 mg/kg) were intravenously injected every three days. When the tumor growth in the mice exceeded ethical limits, the mice were euthanized. All mice were housed under pathogen-free conditions at Zhejiang Chinese Medical University, and the study protocol was approved by the university’s ethics committee.

### Data analysis

Pooled data are presented as the mean ± standard error of mean, unless otherwise indicated. Detailed information on sample sizes, error bars, and statistical analyses can be found in the figure legends. *P* values for statistical comparisons between two groups or multiple groups were calculated using Excel 2021 (Microsoft) and GraphPad Prism (ver. 9.2.0) (GraphPad Software). Data were presented as the mean squared error (SEM). *p* < 0.05 was considered statistically significant.

## Supplementary information

Supplementary figure

Oringinal data

Supplementary table

## Data Availability

The raw ChIP-seq datasets have been deposited in the GEO database under accession numbers [GSE285330].
