# Supplementary material for: KLF7-regulated ITGA2 as a therapeutic target for inhibiting oral cancer stem cells
Source: Cell Death Dis. 2025 May 2;16(1):354. doi: 10.1038/s41419-025-07689-8 (PMC12048542; doi:10.1038/s41419-025-07689-8)
Supplement: Supplementary file 1 — Supplementary figure [file 41419_2025_7689_MOESM1_ESM.pdf]

Supplementary Figure.1

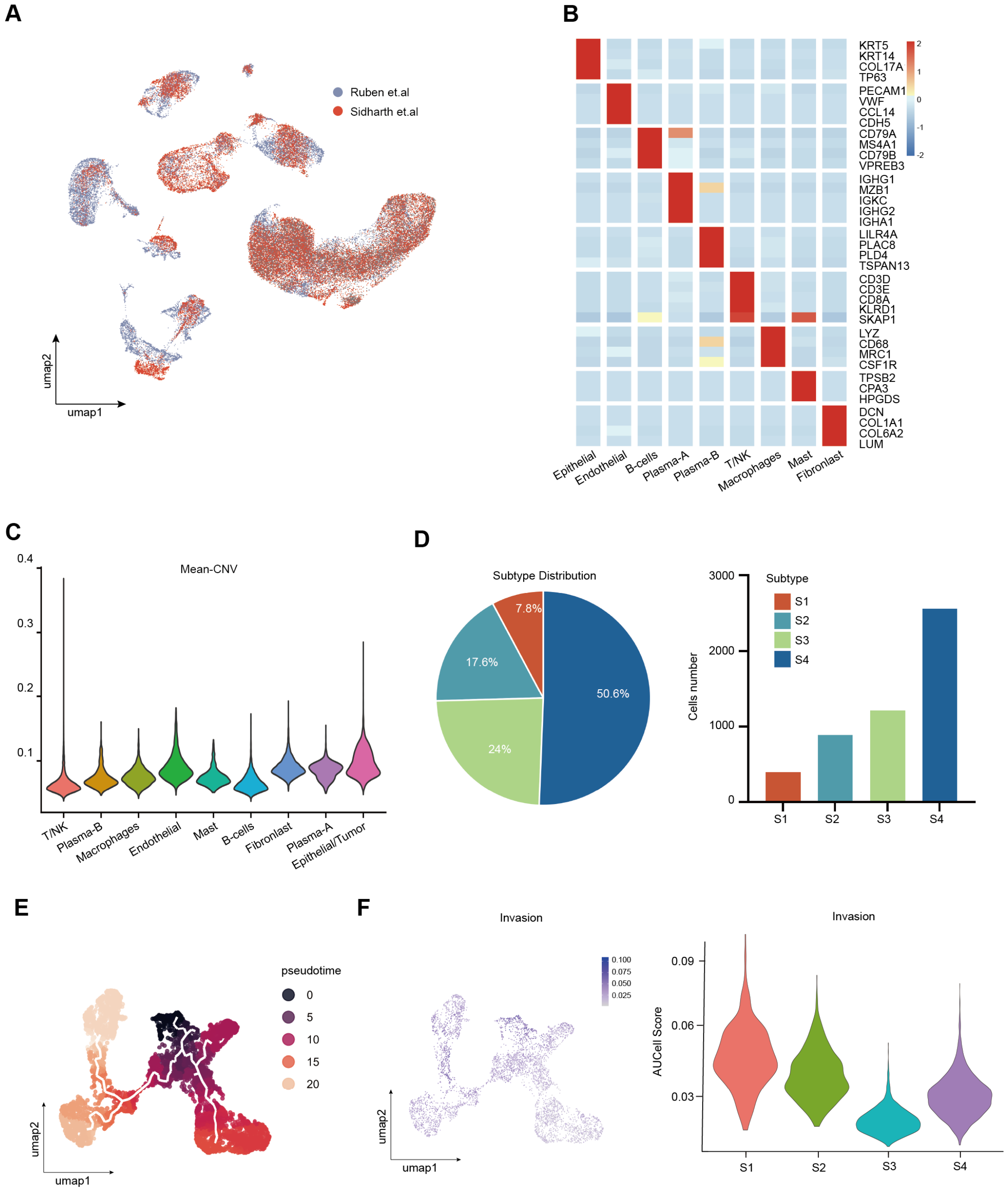

Supplementary Fig.1: Related to Fig.1

A UMAP plot of all cells, colored by OSCC sample source. **B** Heat map showing expression of selected marker genes by all cells assigned to each cell type. **C** Violin plot depicting mean Copy Number Variation (CNV) scores for the different cell types. **D** Distribution and cell numbers of each subtype in Fig. 1B. **E** Psuedotime plot of the subtype of malignant cells. **F** Invasive gene sets activity (AUCell score) in all malignant cells (umap plots, left panel) or per subtype of malignant cells (violin plots, right panel)

Supplementary Fig.2

A

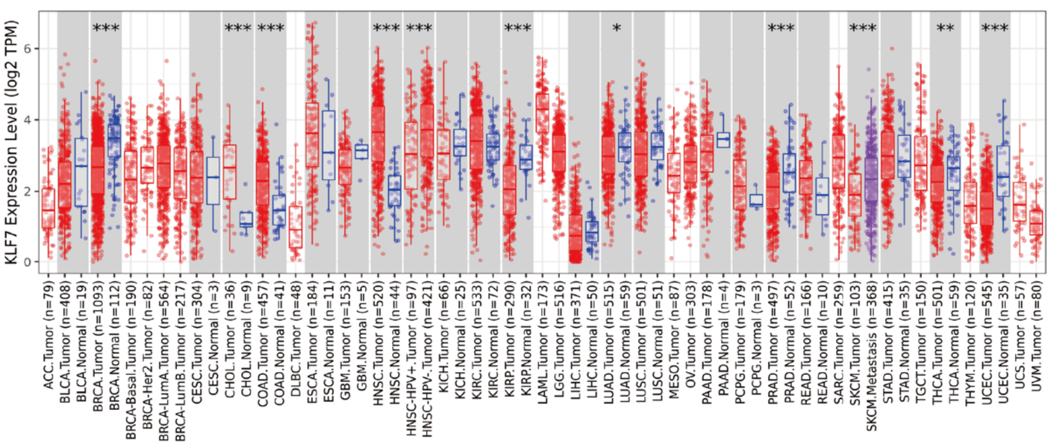

B

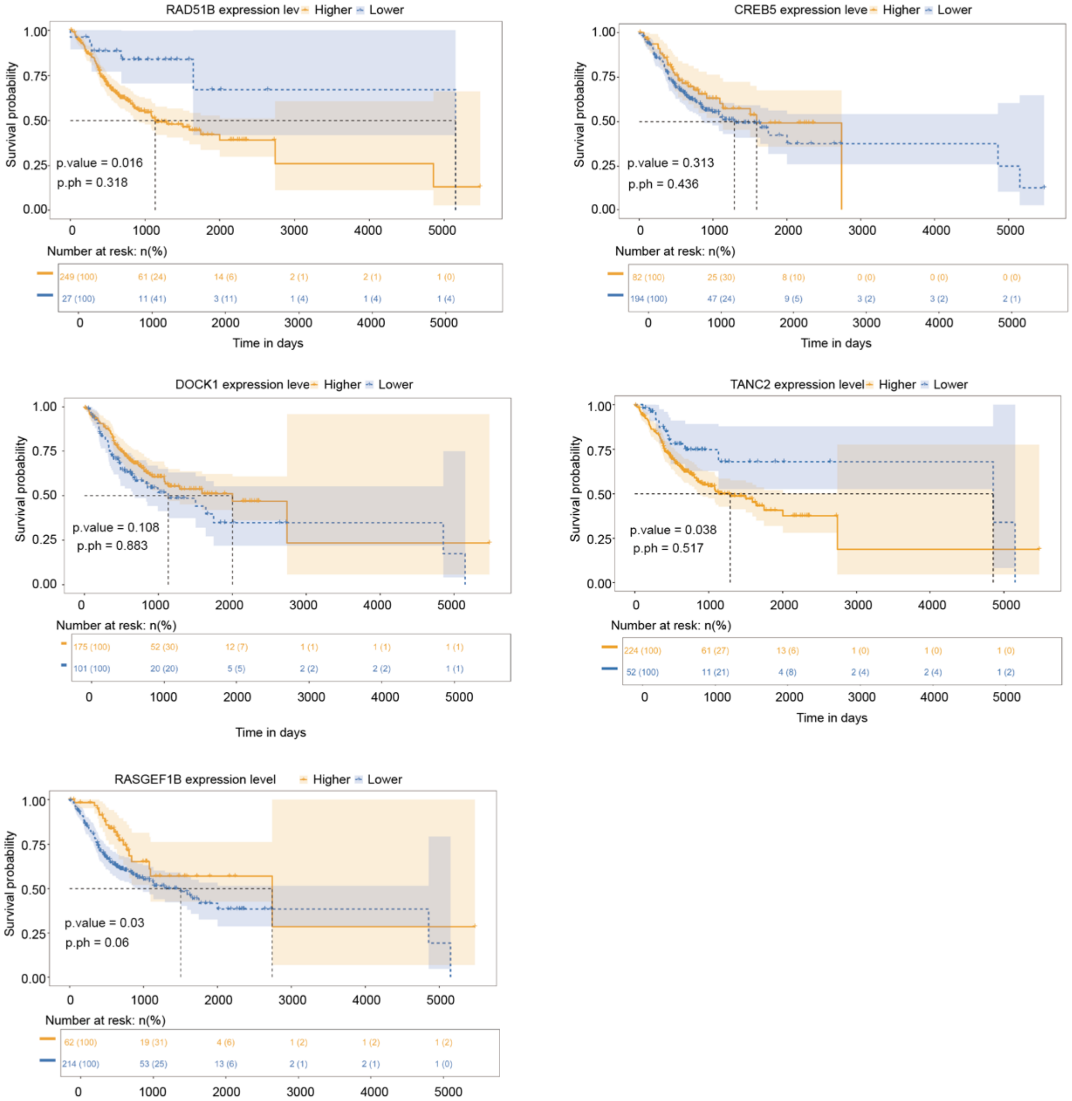

Supplementary Fig.2: Related to Fig.1

A KLF7 expression in pan-cancer in TCGA dataset. **B** Kaplan-Meier curve of RAD51B, CREB5, DOCK1, TANC2, and RASGEF1B in the OSCC TCGA dataset.

Supplementary Fig.3

A

cells number	CAL27 Control	CAL27 shKLF7#1	CAL27 shKLF7#2	HSC3 Control	HSC3 shIKLF7#1	HSC3 shKLF7#2
1000	24/24	24/24	24/24	24/24	24/24	24/24
500	24/24	16/24	15/24	24/24	16/24	13/24
100	18/24	9/24	7/24	19/24	11/24	6/24
50	14/24	4/24	3/24	11/24	5/24	3/24
CSC frequency 95% CI	1/65 (1/94-1/45)	1/310 (1/430-1/224)	1/360 (1/497-1/260)	1/71 (1/102-1/49)	1/283 (1/393-1/203)	1/410 (1/567-1/297)
P.value		P<0.001	P<0.001		P<0.001	P<0.001

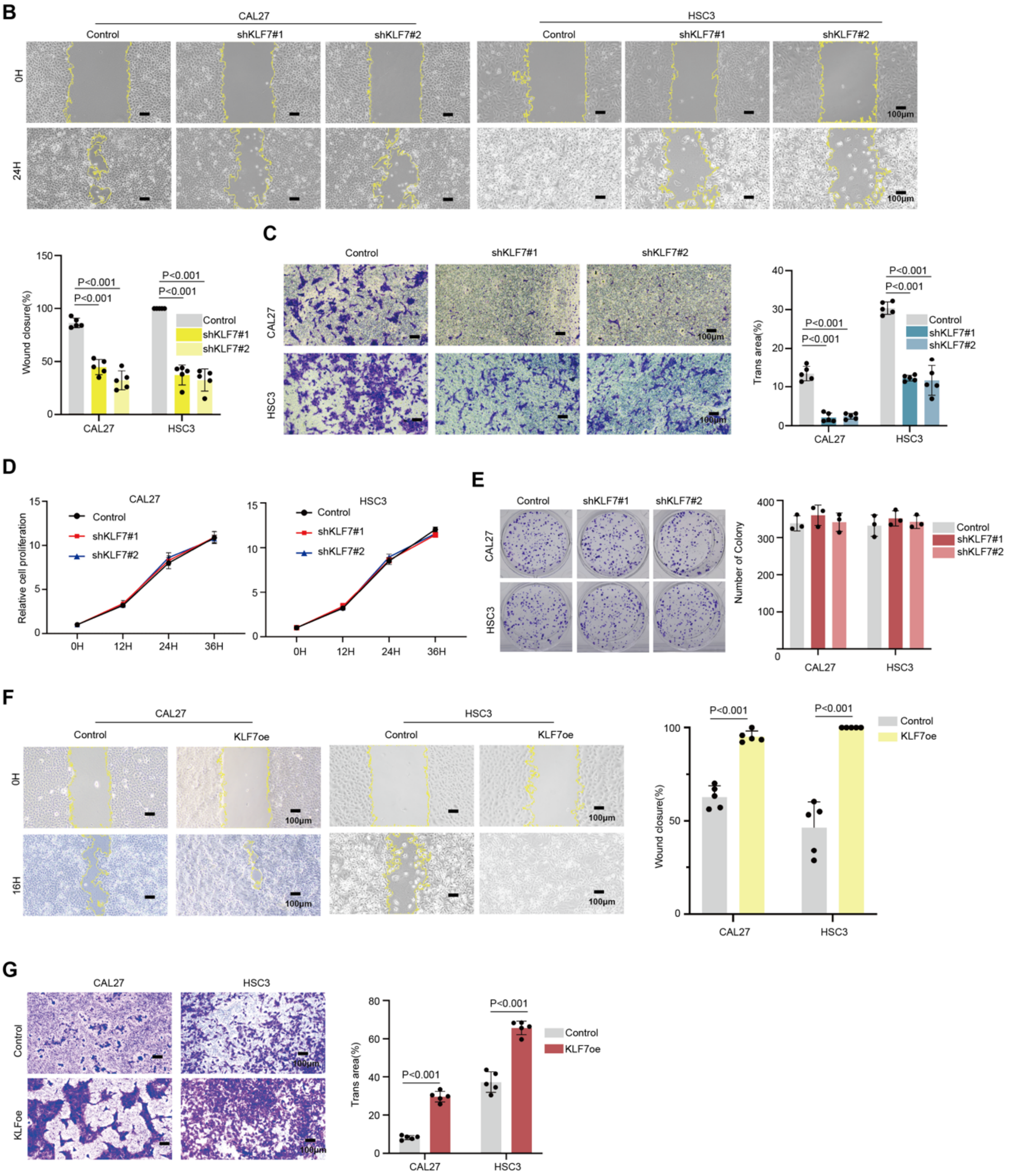

Supplementary Fig.3: Related to Fig.2

A Statistical result of limiting dilution assay using CAL27 cells and HSC3 cells upon KLF7 knockdown. (Statistical significance was determined using the t-statistic approach). **B, C** Wound healing assay (top) and Transwell migration assay (bottom) reveal attenuated migratory capacity in KLF7-silenced cells;

data are presented as mean ± SD from five independent experiments, and statistical significance was determined using Student's t-test. Scale bar, 100μm. **D, E** CCK-8(left)and colony formation assay(right) reveal that KLF7 knockdown does not influence cell proliferation; data are presented as mean ± SD from six independent experiments, and statistical significance was determined using Student's t-test. **F, G** Wound healing assay (top) and Transwell migration assay (bottom) reveal enhanced migratory capacity in KLF7-overexpressed cells; data are presented as mean ± SD from five independent experiments, and statistical significance was determined using Student's t-test. Scale bar, 100μm.

Supplementary Figure.4

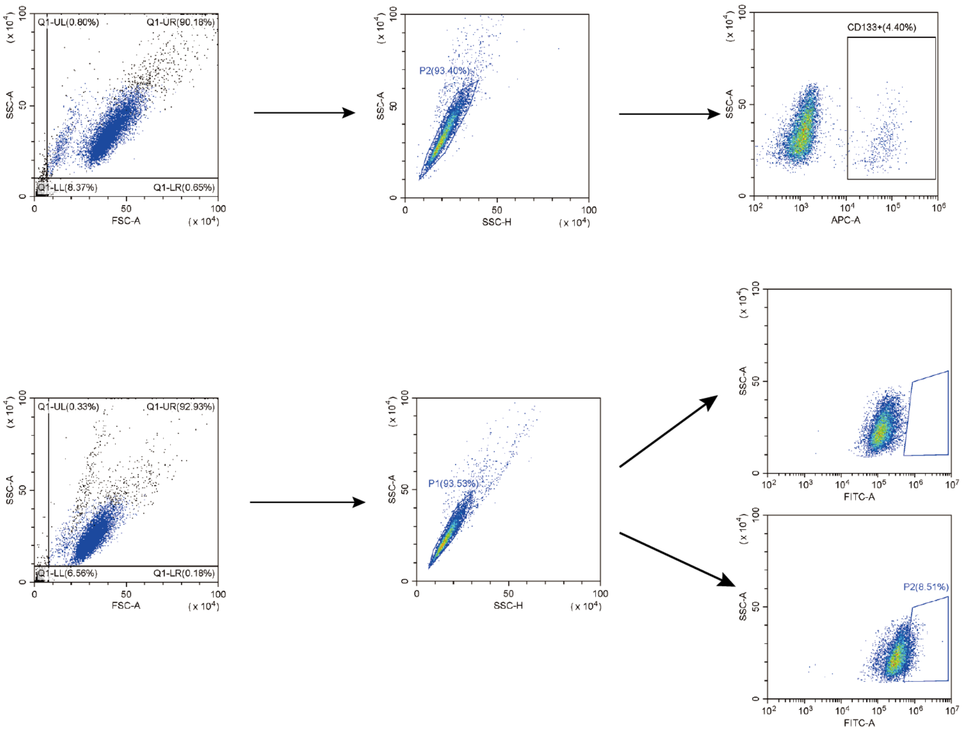

Supplementary Fig.4: Figure exemplifying the gating strategy in flow cytometry

Gating strategy: cells were first gated using FSC and SSC, the dead cells that separated from the major population were removed in this step.1,2. Further on, cells from P1 were tested for CD133 and ALDH activity. Cells with a higher ALDH signal than the DEAB group were defined as the ALDH+ population.

Supplementary Figure.5

A

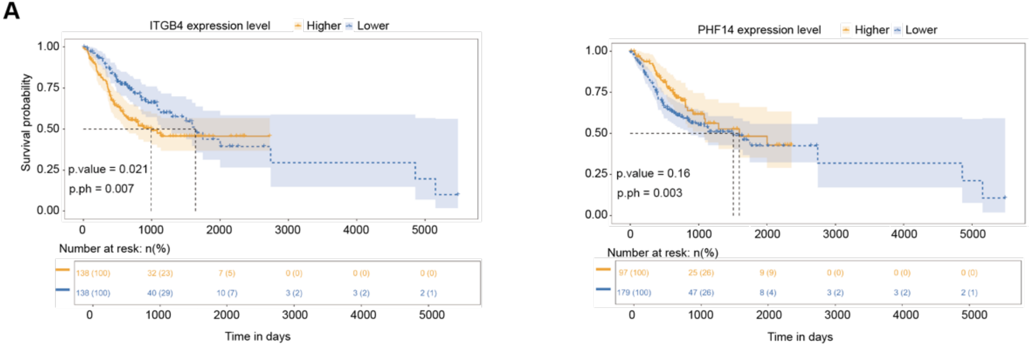

B

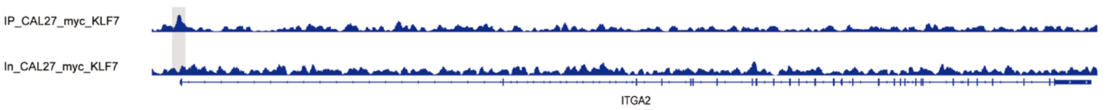

Supplementary Fig.5: Related to Fig.3

A Kaplan-Meier curve of ITGB4 and PHF14 in the OSCC TCGA dataset. **B** The ChIP-seq tracks of KLF7 binding on ITGA2.

Supplementary Figure.6

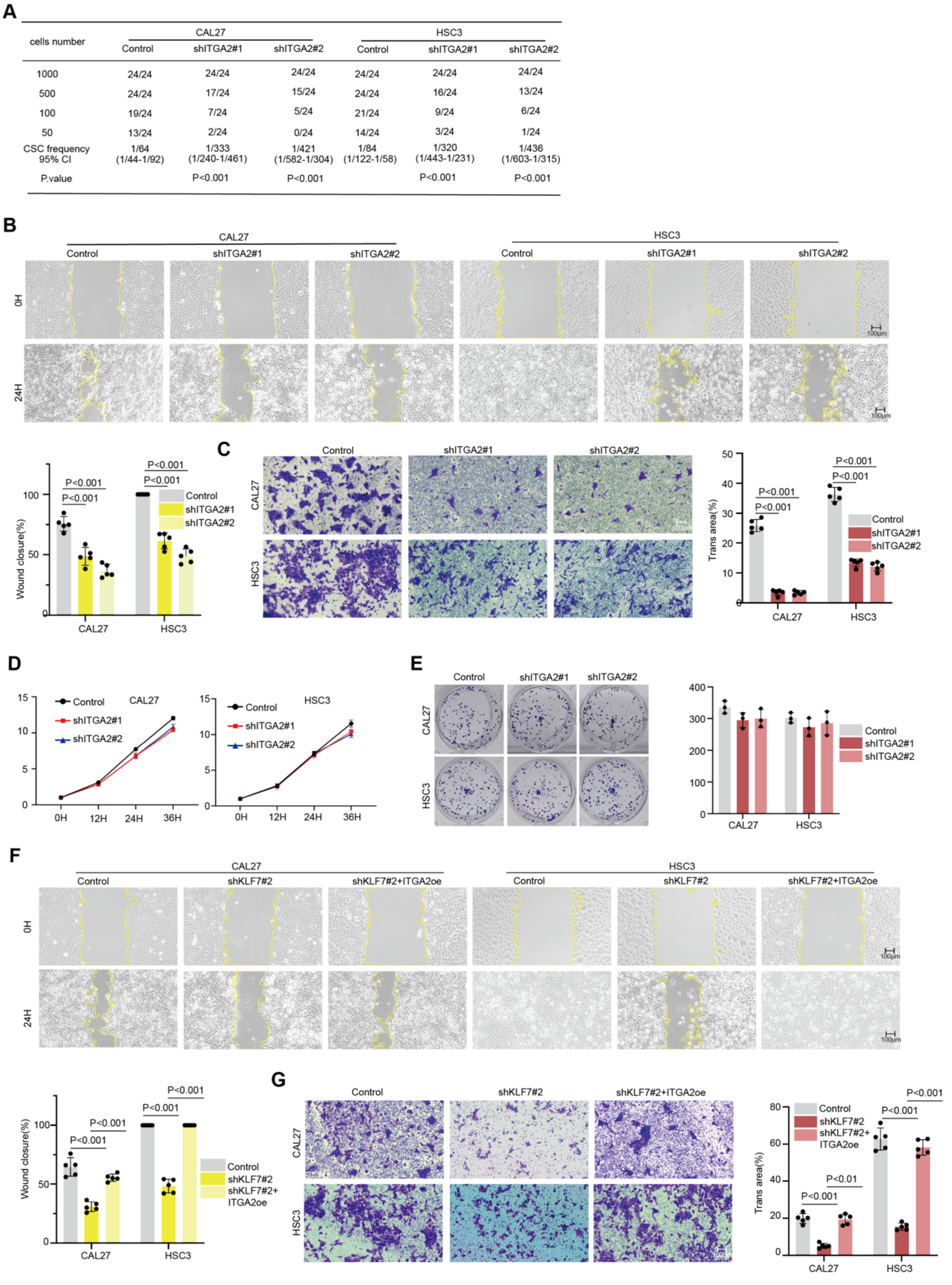

cells number	CAL27 Control	CAL27 shITGA2#1	CAL27 shITGA2#2	HSC3 Control	HSC3 shITGA2#1	HSC3 shITGA2#2
1000	24/24	24/24	24/24	24/24	24/24	24/24
500	24/24	17/24	15/24	24/24	16/24	13/24
100	19/24	7/24	5/24	21/24	9/24	6/24
50	13/24	2/24	0/24	14/24	3/24	1/24
CSC frequency 95% CI	1/64 (1/44-1/92)	1/333 (1/240-1/461)	1/421 (1/582-1/304)	1/84 (1/122-1/58)	1/320 (1/443-1/231)	1/436 (1/603-1/315)
P.value		P<0.001	P<0.001		P<0.001	P<0.001

Supplementary Fig.6: Related to Fig.6

A Statistical result of limiting dilution assay using CAL27 cells and HSC3 cells upon ITGA2 knockdown. (Statistical significance was determined using the t-statistic approach). **B, C** W Wound healing assay (top) and Transwell migration assay (bottom) reveal attenuated migratory capacity in ITGA2-silenced cells; data are presented as mean ± SD from five independent experiments, and statistical significance

was determined using Student’s t-test. Scale bar, 100μm. **D, E** CCK-8(left)and colony formation assay(right) reveal that ITGA2 knockdown does not influence cell proliferation; data are presented as mean ± SD from six independent experiments, and statistical significance was determined using Student’s t-test. **F, G** Wound healing assay (top) and Transwell migration assay (bottom) reveal that the overexpression of ITGA2 rescues the impaired migratory capacity of KLF7-silenced cells.; data are presented as mean ± SD from five independent experiments, and statistical significance was determined using Student’s t-test. Scale bar, 100μm.
