# Supplementary figures and images for: KLF7-regulated ITGA2 as a therapeutic target for inhibiting oral cancer stem cells
Source: Cell Death Dis. 2025 May 2;16(1):354. doi: 10.1038/s41419-025-07689-8 (PMC12048542; doi:10.1038/s41419-025-07689-8)

## Slide 1
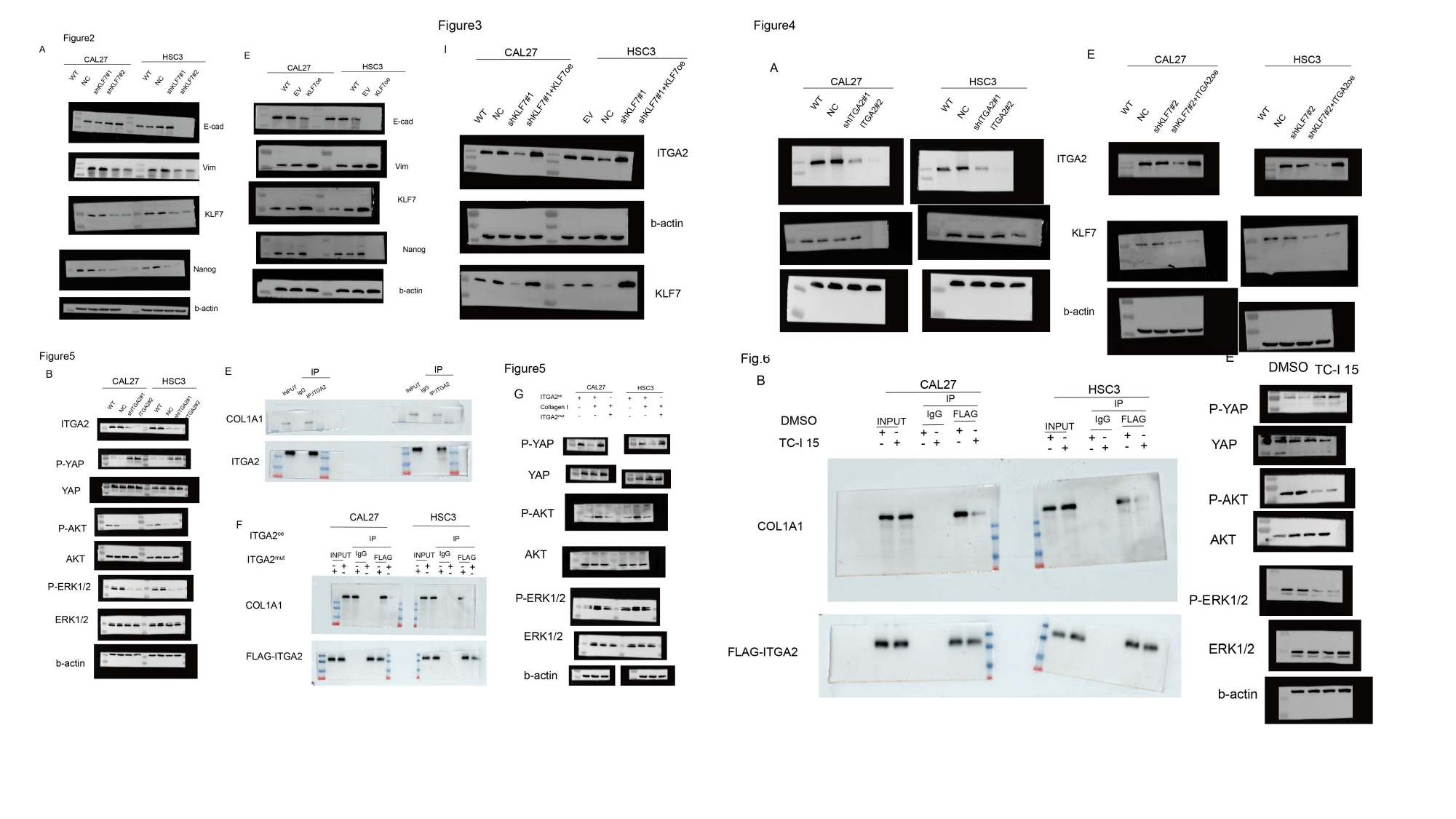

Supplement: Supplementary file 2 — Oringinal data [file 41419_2025_7689_MOESM2_ESM.pptx]
